# Pyrethroid genetic resistance in the dengue vector (*Aedes aegypti*) in Posadas, Argentina

**DOI:** 10.3389/fpubh.2023.1166007

**Published:** 2023-04-27

**Authors:** Jessica V. Fay, Sonia L. Espinola, María V. Boaglio, María J. Blariza, Karen Lopez, Fabian Zelaya, Manisha A. Kulkarni, Carina F. Argüelles, Julian A. Ferreras, Marcos M. Miretti

**Affiliations:** ^1^Laboratorio GIGA, Facultad de Ciencias Exactas, Químicas y Naturales, Instituto de Biología Subtropical, Universidad Nacional de Misiones—Consejo Nacional de Investigaciones Científicas y Técnicas, Posadas, Misiones, Argentina; ^2^Centro de Zoonosis, Secretaría de Planeamiento Ambiental, Ministerio de Salud de Misiones, Posadas, Argentina; ^3^School of Epidemiology and Public Health, University of Ottawa, Ottawa, ON, Canada

**Keywords:** *Aedes aegypti*, pyrethroid resistance, knock-down resistance (kdr), arboviral vector, dengue vector surveillance, vector abundance

## Abstract

Pyrethroids are extensively used to control adult populations of the arboviral vector *Aedes aegypti*, raising concerns regarding the increasing frequency and distribution of insecticide resistance mutations (kdr: knock-down resistance) in the voltage-gated sodium channel gene (*Nav*). The widespread use of pyrethroids imposes a threat to the success of mosquito control and the environment. In this study, we investigated the presence of two kdr mutations (V1016I and F1534C) in the *Nav* gene and their distribution across four neighborhoods in Posadas, Argentina, with different *Ae. aegypti* abundance and contrasting socioeconomic status (SES). Alleles at each locus were interrogated using TaqMan SNP genotyping assays in DNA extracted from adult females collected in a longitudinal study. We report the presence of both pyrethroid resistance alleles (kdr 1016I = 29.08%; kdr 1534C = 70.70%) among adult females. The frequency of combined kdr genotypes reveals that approximately 70% of local adult females have enhanced resistance to pyrethroids. Both, the proportion of resistant adult females (with at least one kdr allele in each locus) and *Ae. aegypti* abundance showed an uneven distribution between neighborhoods with different SES (*p* < 0.001). In high-SES neighborhoods, we found more mosquitoes and a higher frequency of pyrethroid resistance, possibly as a consequence of different public health interventions, social habits, and insecticide use. This is the first report of kdr mutations in *Ae. Aegypti* in the northeast region of Argentina. Our results focus on the need for within-population (city) distribution analyses of kdr mutations and highlight the relevance of incorporating insecticide resistance monitoring within the Integrated Vector Management initiative.

## Introduction

*Aedes aegypti*, the primary vector of arboviral diseases such as Zika, dengue, yellow fever, and chikungunya, is unquestionably adapted to anthropogenic environments, and it is one of the most threatening species to human health worldwide (1). Hundreds of millions of dengue infections every year inflict a huge public health burden in tropical and subtropical countries, a consequence of an ever-expanding range of arboviral infections and vector populations during the last 50 years (2–4). During the 2019–2020 dengue epidemic, Misiones Province, located in the northeast region of Argentina (NEA), registered most of the cases (5). An arboviral survey during 2019–2021 confirmed the circulation of three dengue virus serotypes in the main districts of Misiones, but Zika or chikungunya viruses were not found (6). The recent report of more than 2,000 chikungunya cases 10 miles away across the Paraná River (7) and the circulation of all dengue serotypes in the bordering countries (8), represent major risk factors in Misiones Province considering the high local *Aedes aegypti* larvae index, egg counts, and active fed females documented year-round (9).

Arboviral disease prevention and mitigation strategies are primarily addressed to control the vector by insecticide spraying and eliminating breeding sites and, more recently, by incorporating biotechnologically modified vectors. The rather intense and widespread use of pyrethroid insecticides led to the development of insecticide resistance due to the dissemination of genetic resistance in vector populations worldwide (10–12). Pyrethroids interrupt the mosquito's nerve function by binding to the voltage-dependent sodium channel proteins (11). Its neurotoxic activity is diminished by non-synonymous knock-down-resistant (kdr) mutations introducing amino acid changes in the pyrethroid target protein (13, 14), a predominant mechanism of genetic insecticide resistance.

Several kdr mutations have been identified in *Ae. aegypti*, two of them consistently associated with pyrethroid resistance, i.e., a Val-to-Ile amino acid change at protein position p.1016 (kdr V1016I) and the Phe-to-Cys amino acid change at p.1534 (kdr F1534C) (1, 15). Studies validated the association between these mutations and permethrin resistance (14, 16), showing that at both positions, homozygous kdr genotypes (II/CC) are more resistant than the wild-type homozygous kdr genotypes (VV/FF), with heterozygous genotypes displaying a range of intermediate resistance phenotypes (1, 17, 18). Reports also showed that the kdr V1016I mutation may synergize with the kdr F1534C in generating more resistant phenotypes (1, 15, 17).

Countries in the Americas, i.e., Venezuela, Mexico, USA, Costa Rica, and Brazil, reported increasing kdr V1016I and F1534C frequencies during the last decade, with kdr alleles reaching fixation in some cities (1, 11, 19–21). However, the distribution of kdr alleles within cities remains to be explored, particularly in different demographic and socio-economic settings.

Two insecticide sensitivity studies in Argentina showed incipient resistance in *Ae. aegypti* larvae and adults (22, 23); however, assessments of vector genetic resistance to pyrethroids have not been included in the local Integrated Vector Management programs. The presence, frequency, and distribution of pyrethroid kdr alleles using direct genotyping methods have not been reported in NEA to date. Within a range of 600 miles, only a Brazilian extensive study reported high kdr allele frequencies (11).

Monitoring frequencies of kdr alleles as an indicator of genetic resistance in *Ae. aegypti* populations gives public health authorities a direct assessment tool to evaluate the efficiency of control strategies and means to guide targeted interventions. In the context of the local entomological and arboviral vigilance program, we investigated the presence and distribution of alleles associated with resistance to pyrethroids, demonstrating for the first time the presence of kdr mutations in *Ae. aegypti* populations in Misiones, Argentina. Resistant (kdr) genotypes showed a non-random spatial distribution between contrasting socioeconomic status (SES) districts, distinguishing neighborhoods with increased pyrethroid resistance degree and *Ae. aegypti* abundance.

## Methods

### Experimental setting, study site, and biological material

The collection of adult specimens of *Ae. aegypti* was carried out as part of an international research collaboration network (RADAM-LAC) to investigate determinants of *Ae. aegypti* density at the household level, including factors related to household characteristics (i.e., crowding, water and waste management, and protection against mosquitoes) (9). Sampling was performed on a household basis simultaneously in three cities, i.e., Manta (Ecuador), Bage (Colombia), and Posadas (Argentina), following the same experimental design and methodology (9).

**Figure 1:**
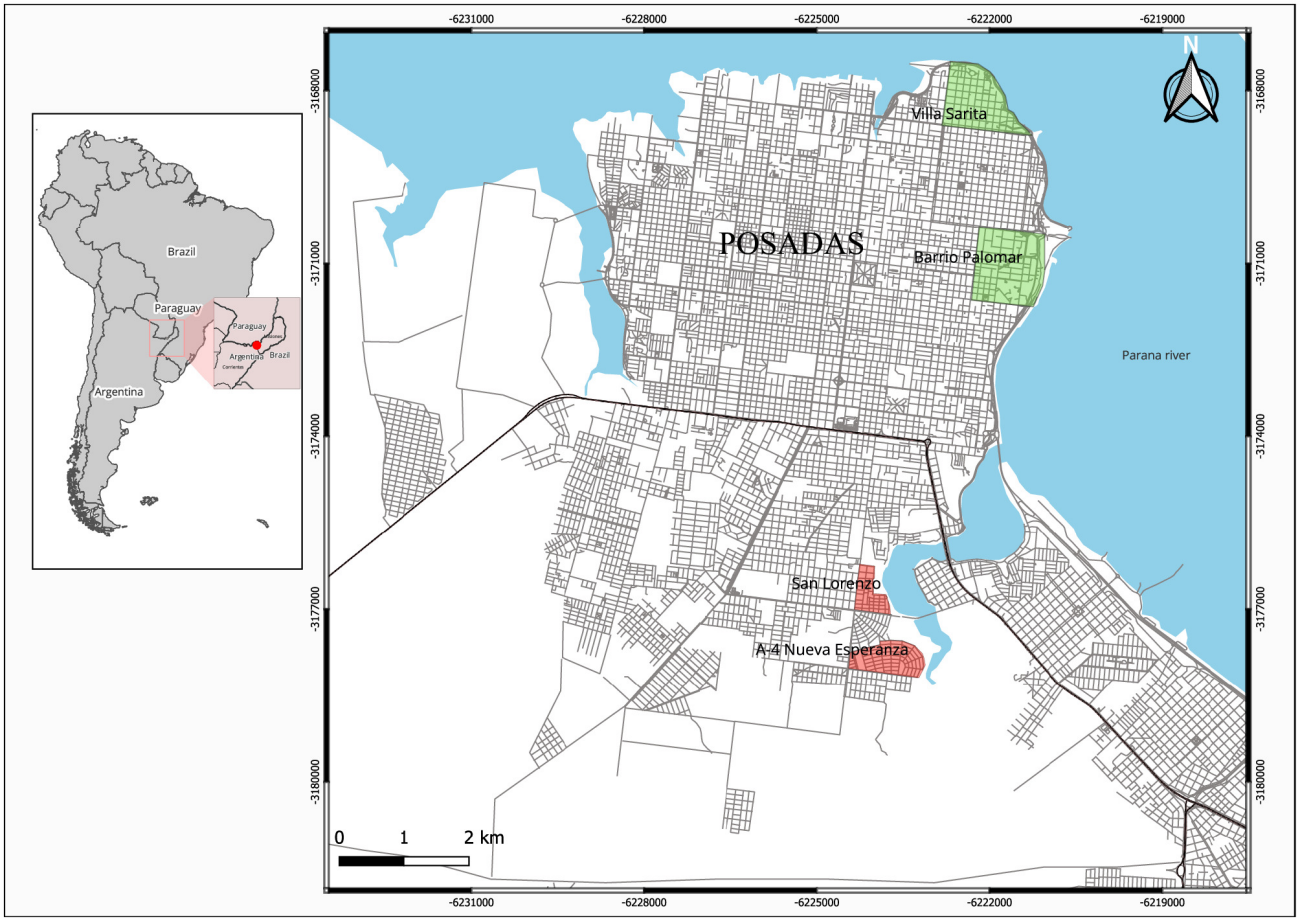
Distribution of *Aedes* collection sites on low (red) and high (green) socio-economic status (SES) neighborhoods in Posadas, Argentina.

Adult *Ae. aegypti* were collected from 372 houses during a longitudinal study (January to December 2019) carried out in Posadas, NEA (27°22′00″S 55°53′49″O), a city of 4,00,000 inhabitants on the banks of the Paraná River, 120 m.o.s.l., with recurrent dengue epidemics. Four neighborhoods were grouped according to their population density, household crowding, household wealth, type of housing, and public services availability (water and sewage): two neighborhoods into “high” socioeconomic status (SES), privileged longstanding residential areas close to the city center [Villa Sarita (VS) and Barrio Palomar (BP)]; and two into “low” SES, less favored recent housing areas in the periphery [San Lorenzo (SL) and Nueva Esperanza (NE)] (Figure 1). Three adult mosquito traps (including one BG-Sentinel 2, one CDC miniature light trap, and one resting trap) were set in each of the 93 selected houses per neighborhood, and mosquitoes were also aspirated from indoor/outdoor surfaces using a Prokopack 1419 Aspirator [for details, see (9)]. Adult *Ae. aegypti* were sorted (males, fed females, and unfed females) and dissected, one piece individually stored at −20°C and the other part placed in pools of 20 individuals for subsequent molecular analyses. For the kdr assessment, 25 adult *Ae. aegypti* females were randomly selected from adult mosquitoes captured in different houses within each neighborhood during the highest prevalence sampling period (November–April).

### Identification of knock-down resistance mutations

Individual DNA that was isolated (Wizard Genomic DNA Purification Kit, Promega) from 100 dissected adult *Ae. aegypti* (50 from each SES group) was used as a template for a direct genotyping of two kdr mutations in the *Nav* gene (kdr V1016I and kdr F1534C) using validated TaqMan SNP assays (Thermo Fisher) as published in Melo Costa et al. (11). Positive DNA controls for resistant genotypes were kindly provided by Dr. Adriana E Flores, UANL, Mexico. A separate, non-template reaction was used as a negative control. Primer and probes sequences and reaction assays are detailed in Supplementary Table 1.

### Statistical analyses

Allelic and genotypic frequencies were estimated by direct count, individually at each position (i.e., *Nav* p.1016: VV, VI, II; and *Nav* p.1534 FF, FC, CC). The frequency of combined genotypes, V1016I +F1534C, was also estimated (i.e., VV/FF, VV/FC, VV/CC, VI/FF, VI/FC, VI/CC, II/FF, II/FC, II/CC), considering that they are physically very close in the *Ae. aegypti* genome and therefore in the linkage disequilibrium (19, 24). We used the Mann–Whitney U-test to assess the differences in adult females *Ae. aegypti* count in low- vs. high-SES areas and Z-score to evaluate the proportion of resistant and wild-type genotypes in these two areas.

## Results

The 2019 longitudinal study assessed 372 houses in four neighborhoods (two high and two low SES) in Posadas, Argentina, collecting mosquitoes indoors and in the peridomicile area using four validated trapping methods in each site. In total, we collected 4,391 *Culex* spp. and 1,020 *Ae. aegypti* adult mosquitoes. High-SES neighborhoods, Villa Sarita + Barrio Palomar (VS+BP), accounted for a higher number of adult *Ae. aegypti* per household and *Ae. aegypti* abundance, showing significant differences in the proportion of captured *Ae. aegypti* (z-score: −16.48225, p < 0.001) (Table 1).

**Table 1:** Adult *Ae. aegypti* captured in low (NE+SL) and high (VS+BP) socioeconomic status (SES) neighborhoods.

	**Neighborhood**	***Ae. aegypti* abundance**	**Number of adult Aedes per household**
Low SES	NE	226	2.43
	SL	176	1.89
	*Total*	**402** ^*^	***2.16***
High SES	VS	313	3.36
	BP	305	3.28
	*Total*	**618** ^*^	***3.32***

^*^Significantly different p < 0.01. The bold values indicate totals or sub totals.

TaqMan genotyping individual mosquitoes for two kdr mutations, V1016I (n = 98) and F1534C (n = 99), showed a high frequency of pyrethroid resistance alleles in local *Ae. aegypti* populations, whereas kdr V1016I mutation was present in 49% of analyzed adults, kdr F1534C was observed in 92% of individuals. Frequencies of the resistant alleles at both positions were substantially different (kdr 1016I = 0.2908; kdr 1534C = 0.7070). For each position, all three genotypes (i.e., *Nav* p.1016: VV, VI, II; *Nav* p.1534 FF, FC, CC) were identified in the assayed sample. The resistant homozygous kdr 1534C genotype (CC) was the second most frequent. Observed genotypic frequencies (Table 2) are in Hardy–Weinberg equilibrium.

**Table 2:** Frequency of knock-down-resistant (kdr) alleles and genotypes found at *Nav* p.1016 and *Nav* p.1534 positions in *Aedes aegypti* from Posadas, Argentina.

**kdr position**	**Allele**	**Frequency (%)**	**Genotype**	**Frequency (%)**
1016	**V**	70.91	**VV**	51.02
			**VI**	39.80
	**I** ^*^	29.08	**II**	9.18
				*n = 98*
1534	**F**	29.29	**FF**	8.08
			**FC**	42.42
	**C** ^*^	70.70	**CC**	49.49
				*n = 99*

^*^Knock-down-resistant alleles at Nav p.1016 and Nav p.1534C.

The combined genotypes considering both the 1016 and 1534 *Nav* sites and their frequencies found in this study are listed in Table 3. Combined genotypic frequencies showed that 92.63% of all individuals carried at least one resistant allele and 50% at least one resistant allele in each position, i.e., VI/FC, VI/CC, and II/CC (Table 3). VI/FF, II/FC, and II/FF genotypes were not observed.

**Table 3:** Frequency (%) of combined kdr genotypes (V1016I + F1534C) in *Aedes aegypti* from Posadas, Argentina.

Combined genotypes^*^	**Whole sample (*N* = 95)**	**Low-SES neighborhood (*N* = 50)**	**High-SES neighborhood (*N* = 45)**
V/V/FF	7.37	14.00	0.00
VV/FC	23.16	26.00	20.00
VV/CC	20.00	26.00	13.33
VI/FC	20.00	14.00	26.67
VI/CC	21.05	16.00	27.67
II/CC	8.42	4.00	13.33
		**0.34** ^**^	**0.67** ^**^

^*^VI/FF, II/FC–II/FF genotypes absent.

^**^Significant different (p < 0.001). The bold values indicate totals or sub totals.

The genotype counts in low- and high-SES neighborhoods were not proportional to their distribution in the whole sample (*p* < 0.001, Table 3). There was a higher prevalence of genotypes carrying at least one resistant allele in both positions (VI/FC, VI/CC, and II/CC) in mosquitoes collected from high-SES (67%) compared with low-SES neighborhoods (34%). In addition, the susceptible double homozygous genotype (VV/FF) was absent in high-SES districts but represented 14% of mosquitoes collected from the low-SES districts.

## Discussion

The arboviral vector mosquito *Aedes aegypti* is a major threat to human health worldwide. Its control represents a vital challenge for public health authorities in subtropical and tropical countries. Genetic resistance to insecticides is well documented in several species, including *Ae. Aegypti*, and has been largely validated in physiological and molecular studies (8, 22), showing that mutations in the *Nav* gene are responsible for weakening the pyrethroid knock-down efficacy. Investigations have extensively portrayed the effect of kdrV1016I and kdrF1534C mutations and their fluctuating distribution in the Americas (1, 11, 25).

Here, we report for the first time the presence and distribution of *Ae. aegypti* kdr mutations in the northeast region of Argentina. We assessed the two main pyrethroid resistance markers in adult mosquitoes, kdrV1016I and kdrF1534C, using TaqMan SNP genotyping assays, the most widely used genotyping system. Unlike other methods, a TaqMan assay executes direct genotyping, reducing adjustments and additional confirmatory reactions to non-specific amplifications (17).

In total, 92.63% of all tested mosquitoes had at least one resistant allele. The high kdr allelic frequencies observed in this study, particularly in kdr F1534C (70.7%), derived from the predominance of homozygous resistant genotypes identified in approximately 50% of mosquitoes (Table 2). In the absence of previous *Ae. aegypti* kdr data gathered in Argentinian nearby regions (within 600 miles), our report gains relevance as a reference for future studies, particularly to assist local vector control strategies. The closest kdr survey reported was carried out in a Brazilian border city 210 miles away, showing even higher frequencies in both kdr mutations, i.e., V1016I = 70% and F1534C = 91% (11).

Co-occurrence of kdr mutations (V1016I+F1534C) presenting at least one copy of the resistant allele at each kdr site is common, reaching up to 90% in Central and South American districts (1), possibly as an outcome of the intense use of pyrethroids. According to the hypothesis of the synergistic contribution of kdr V1016I + kdr F1534C, homozygous resistant genotypes at both positions, i.e., Nav.p1016/p.1534 (II/CC), are more permethrin resistant than the VI/CC, and these, in turn, more than the VV/CC (15), demonstrating the individual contribution of kdr alleles. Our data show that VI/CC and II/CC genotypes represent ~30% of combined genotypes observed in Posadas' neighborhoods. Furthermore, almost 50% of the assayed adult *Ae. aegypti* presented at least one kdr allele in both loci, indicating a currently extensive degree of pyrethroid resistance. Combined genotypes II/FC and II/FF were not found in Posadas, in line with their exceptionally low frequency (0.0004) reported by the comprehensive survey conducted in 123 Brazilian cities (11). Hernandez et al. (1) suggested that the low frequency of genotypes II (Nav p.1016) could be associated with their subsequent evolution after F1534C, as well as with a lower fitness of these genotypes. Selection pressure due to the extensive use of pyrethroids is also a likely scenario.

Examining the distribution of combined resistant genotypes carrying at least one kdr mutation in each position (VI/FC, VI/CC, and II/CC) among neighborhoods showed a significantly increased proportion of kdr genotypes in high-SES areas (box in Table 3). This indicates an increased degree of pyrethroid resistance in privileged SES areas in Posadas, which is confirmed by the absence of a double homozygous susceptible genotype (VV/FF) in this area. Notably, the abundance of *Ae. aegypti* captured in high-SES neighborhoods is also higher (*p* < 0.01, Table 1). Hence, in high-SES neighborhoods, we found not only more mosquitoes but also more resistant ones. The increased *Ae. aegypti* counts alongside prevalent resistant genotypes in high-SES areas may result from two main factors, namely (a) easier access to and more intense and widespread use of household pyrethroid insecticides in high-SES districts and (b) comparative success of usual vector control strategies mostly associated with the elimination of breeding sites and debris removal actions in low-SES areas (corroborated by City Public Health intervention frequency). In line with this, the resistance to insecticides for domestic use and the selection pressure associated with this extensive practice have already been demonstrated in *Ae. aegypti* (11, 26, 27). In Brazil, a non-random distribution of kdr genotypes among cities, with the absence of the wild-type in localities where chemical control inside houses played a fundamental role, was observed (11). Also, an increased frequency of kdr V1016I mutation was associated with the use of surface pyrethroid aerosols in homes in Mexico (26). Extensive chemical control operations have been performed in the north of Argentina since 1998, and systematic actions began in 2003 in Misiones, Argentina. In recent years, local health authorities have implemented controls with type 1 pyrethroids [(1-RS)-cis-trans permethrin].

In this context, the consideration of *Ae. aegypti* abundance without information on the frequency of the pyrethroid resistance alleles in vector populations would have led to making inappropriate decisions on vector control management in high SES. Crucially, the available kdr data offer the opportunity to redirect strategies and reduce the amount and/or type of pesticide spraying by a precision-guided use of insecticides within cities. Considering the lack of regional kdr data associated with *Ae. aegypti* abundance, this study contributes to the first reference dataset for local vector vigilance and insecticide resistance management programs. Continuous evaluation of permethrin resistance across neighborhoods by monitoring V1016I + F1534C kdr mutations and insecticide bioassay testing will allow the analysis of the spatiotemporal evolution of the resistance phenomenon, a chance to effectively plan localized control strategies including the use of alternate insecticides and non-chemical interventions.

This study provides evidence regarding two premises that have been poorly addressed that represent key issues in the design and implementation of vector control strategies. First, (a) between-neighborhood (subpopulations) differences in kdr genotypic frequencies: studies mostly evaluate kdr data from each city (sample), comparing results between cities in country-wide or statewide studies. In addition to the actual relevance of those analyses, here we found skewed kdr genotype frequencies between two groups of neighborhoods within the same city. Second, (b) the complex relationship among *Ae. aegypti* abundance, pyrethroid resistance, and socio-economic (cultural) determinants. Both require further experimental analyses and monitoring in different eco-epidemiological settings.

Frequency estimates are based on genotyping 100 *Ae. aegypti* adults, representing only ~10% of the captured samples. This could be interpreted as a limitation of this study; however, increasing the sample size would not imply significant changes as allele frequencies at both positions are moderate to high, and genotypic frequencies are under Hardy–Weinberg equilibrium. Notwithstanding this, the spatial expansion of the sampling by including all neighborhoods in Posadas would generate a more complete understanding of the local *Ae. aegypti* population structuring concerning kdr mutations.

In conclusion, this study demonstrates the relevance of kdr data for precise and focused vector control interventions and public health initiatives to reduce both *Ae. aegypti* populations and insecticide use/misuse to mitigate arboviral disease risks.

## Data availability statement

The original contributions presented in the study are included in the article/Supplementary material, further inquiries can be directed to the corresponding author.

## Author contributions

JFa, KL, FZ, MK, JFe, CA, and MM contributed to the conception and design of the study. JFa, MBo, SE, and MBl performed the laboratory work and analysis of results. JFa, JFe, and MM wrote the first draft of the manuscript. All authors contributed to the revision of the manuscript, read, and approved the submitted version.
