# Supplementary material for: Pyrethroid genetic resistance in the dengue vector (Aedes aegypti) in Posadas, Argentina
Source: Front Public Health. 2023 Apr 27;11:1166007. doi: 10.3389/fpubh.2023.1166007 (PMC10174043; doi:10.3389/fpubh.2023.1166007)
Supplement: Supplementary file 1 [file Table_1.docx]

***Supplementary Information***

Supplementary Table 1. Primer and probe sequences for SNP genotyping *Nav* p.1016I and *Nav* p.1534 kdr loci in *Aedes aegypti* using customized TaqMan SNP Genotyping Assays (TermoFischer) (11).

| **Nav site** | **Assay ID** | **Variation** | **Primers** | **Probes** |
| --- | --- | --- | --- | --- |
| 1016 | AHS1DL6 | GTA/ATA | For: CGTGCTAACCGACAAATTGTTTCC | Val: VIC-CCCGCACAGGTACTTA-FAM |
|  |  | (Val/Ile) | Rev: GACAAAAGCAAGGCTAAGAAAAGGT | Ile: FAM-CCGCACAGATACTTA-NFQ |
| 1534 | AHWSL61 | TTC/TGC | For: TCGCGAGACCAACATCTACATG | Phe: VIC-AACGACCCGAAGATGA-NFQ |
|  |  | (Phe/Cys) | Rev: GATGATGACACCGATGAACAGATTC | Cys: FAM-ACGACCCGACGATGA-NFQ |

*Two TaqMan SNP genotyping systems were individually assayed. PCR cycling conditions included 45 cycles consisting of DNA denaturation step (95 °C for 15 s), primer and probe annealing followed by DNA polymerization step (60 °C for 1 min). The genotypes were obtained by the online sofware Genotype Analysis Module (Applied Biosystems, TermoFischer).*
